# Tracheobronchomegaly: a case series and review of the literature

**DOI:** 10.3389/fmed.2025.1676083

**Published:** 2025-09-09

**Authors:** Zhenhua Li, Zhen Yang, Yiran Niu, Yan Lei, Jixiang Ni, Rujuan Wang, Fangjing Wen

**Affiliations:** ^1^Department of Respiratory and Critical Care Medicine, The Central Hospital of Wuhan, Tongji Medical College, Huazhong University of Science and Technology, Wuhan, Hubei, China; ^2^School of Medicine, Jianghan University, Wuhan, Hubei, China

**Keywords:** tracheobronchomegaly, Mounier-Kuhn syndrome, interstitial lung disease, respiratory failure, hemoptysis

## Abstract

**Background:**

Tracheobronchomegaly (TBM) is a rare condition characterized by abnormal dilation of the trachea and main bronchi owing to a pathological arrangement of smooth muscle fibers. Early identification and intervention are vital to halting progressive lung damage and improving the quality of life. This study aimed to examine the clinical characteristics, radiological features, and related complications in patients with TBM.

**Methods:**

11 TBM cases were retrospectively identified through a review of chest computed tomography (CT) scans at our hospital between January 2018 and July 2025. We collected sputum or bronchoalveolar lavage fluid from 10 patients, and their clinical, radiological, and pulmonary function data were systematically collected and analyzed to assess disease characteristics and complications.

**Results:**

All 11 patients were male (mean age 79.2 ± 9.1 years). The main symptoms included recurrent infections, hemoptysis, productive cough, and dyspnea. The mean tracheal diameter was 32.4 [interquartile range (IQR):31.4–45.8]mm. Diverticula were present in four cases (36.4%, *n* = 4). Complications included bronchiectasis (45.5%, *n* = 5), chronic obstructive pulmonary disease (36.4%, *n* = 4), respiratory failure (9.1%, *n* = 1), tracheobronchopathia osteochondroplastica (9.1%, *n* = 1), and interstitial lung disease (9.1%, *n* = 1). Pulmonary function tests revealed obstructive (36.4%, *n* = 4), restrictive (27.3%, *n* = 3), and mixed (9.1%, *n* = 1) patterns. One patient died of respiratory failure.

**Conclusion:**

In this study, 11 cases involving a variety complications were analyzed in light of the current literature. TBM should be considered in patients who present with chronic cough, recurrent pulmonary infections, and bronchiectasis.

## Introduction

Tracheobronchomegaly (TBM), also known as Mounier-Kuhn syndrome (MKS) is a rare and possibly congenital condition characterized by abnormal enlargement of the trachea and main bronchi (1). The first documented case was reported in 1932 (2), and fewer than 500 cases have been described in the medical literature to date (3).

The prevalence of TBM is higher in males. Impaired mucociliary clearance and an ineffective cough reflex result in the accumulation of secretions, predisposing individuals to recurrent pulmonary infections and bronchiectasis (4). Consequently, the affected airways are often flaccid and markedly dilated during inspiration but collapse during expiration, which may lead to severe complications, including hemoptysis and respiratory failure. The diagnosis of TBM is primarily based on chest CT and bronchoscopy, which confirm the patient’s condition. In adults, the CT diagnostic criteria include a tracheal diameter > 30 mm and main bronchial diameters > 24 mm and >23 mm for the right and left bronchi, respectively. An increase in the tracheal cross-sectional area beyond 371 mm^2^ in men and 299 mm^2^ in women further supports this diagnosis. This study presents the complications and unusual presentations observed in 11 cases of TBM, as highlighted in relevant studies. Early diagnosis and intervention are crucial for preventing progressive lung damage and improving the quality of life of affected individuals.

## Materials and methods

This study included 11 patients diagnosed with TBM at our hospital between January 2018 and July 2025. Consent was obtained during the diagnostic process, and the diagnosis was confirmed using CT and bronchoscopy. The CT diagnostic criteria for TBM were as follows. In adults, the CT diagnostic criteria included tracheal diameter > 30 mm and main bronchial diameters > 24 mm and >23 mm for the right and left bronchi, respectively. We collected sputum or bronchoalveolar lavage fluid (BALF) from 10 patients; pulmonary function tests (PFTs) were performed in 10 patients unless contraindicated; and clinical, radiological, and pulmonary function data were systematically collected and analyzed to assess disease characteristics and complications. The final data table was analyzed using SPSS 22.0 (IBM Corporation, Armonk, NY, USA) computer program. Acquired data were checked for normality and depending on the results either parametric (Student’s *t*-test, Pearson’s coeffi-cient of correlation) or non-parametric tests (Mann–Whitney U-test, Spearman’s rank correlation) were applied. Results of calculations were rounded to one decimal place. Where information was missing the available data was summarized.

## Results

**FIGURE 1:**
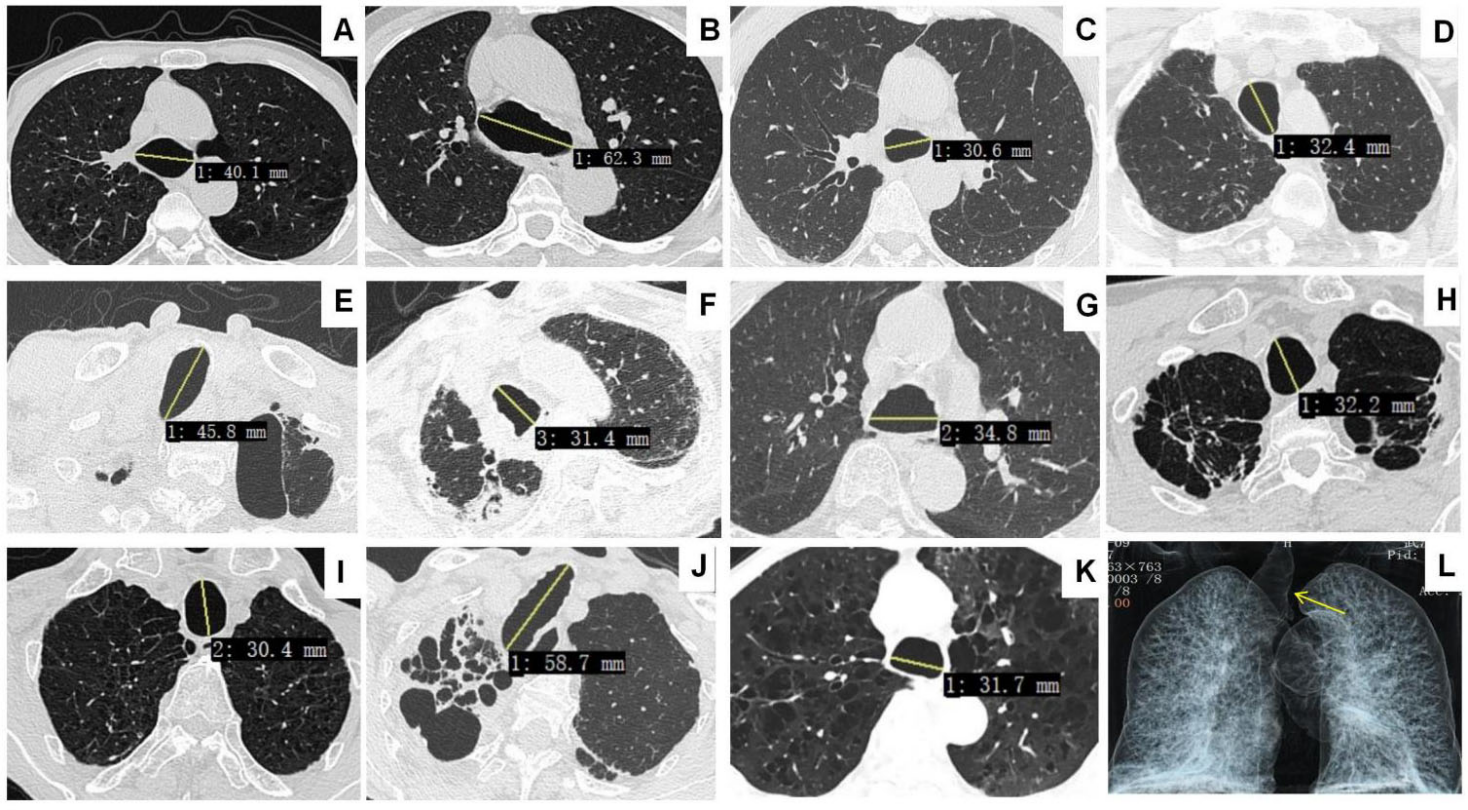
Chest CT showing tracheomegaly in Cases 1–11 **(A–K)**. Airway reconstruction reveals main bronchial dilatation in case 2 (**L**, yellow arrow).

All 11 patients in this study were male, with a mean age of 79.2 ± 9.1 years (range, 61–91 years). The mean tracheal size was 32.4 mm (Figure 1). There was no correlation between patient age and tracheal and/or bronchial diameter. All patients had a history of smoking. Except for one patient (case 6) who was incidentally discovered during a routine physical examination, the major complaints and signs noted in the reports were productive cough (45.5%, *n* = 5), hemoptysis (27.3%, *n* = 3), dry cough (9.1%, *n* = 1), and dyspnea (9.1%, *n* = 1). The final diagnoses included bronchiectasis (45.5%, *n* = 5), chronic obstructive pulmonary disease (COPD) (36.4%, *n* = 4), respiratory failure (9.1%, *n* = 1), tracheobronchopathia osteochondroplastica (TO) (9.1%, *n* = 1), and interstitial lung disease (ILD) (9.1%, *n* = 1). PFTs revealed obstructive (36.4%, *n* = 4), restrictive (27.3%, *n* = 3), and mixed (9.1%, *n* = 1) patterns, as summarized in Table 1. One patient died due to respiratory failure.

**TABLE 1:** Patient characteristics.

Case	Age (year)	Gender	Smoke history (year)	Average tracheal diameter (mm)	Right/left main bronchus diameter (mm)	Chief complaints	Pathogen	Final diagnosis	Outcome
Case 1	77	Male	43	40.1	22.8/21.2	Productive cough	*Streptococcus pneumoniae*	TBM/bronchiectasis	Stable
Case 2	85	Male	62	62.3	32.1/30.3	Productive cough, fever	*Streptococcus pneumoniae*	TBM/pneumonia/TO	Stable
Case 3	61	Male	34	30.6	22.1/20.5	Dry cough	Not determined	TBM/ILD	Stable
Case 4	84	Male	45	32.4	3.6/20.8	Hemoptysis	*Pseudomonas aeruginosa*	TBM/bronchiectasis/COPD	Stable
Case 5	74	Male	37	45.8	28.3/21.7	Dyspnea	*Klebsiella pneumoniae*/*Aspergillus*	TBM/pneumonia/respiratory failure	Death
Case 6	87	Male	32	31.4	20.9/20. 1	Asymptomatic	*Aspergillus*/*Mycobacterium tuberculosis*	TBM/bronchiectasis	Stable
Case 7	91	Male	63	34.8	4.5/22.9	Productive cough, fever	*Aspergillus*/influenza A virus	TBM/pneumonia	Improved
Case 8	68	Male	32	32.2	23.1/22.0	Productive cough, fever	*Klebsiella pneumoniae*	TBM/COPD/lung abscess	Improved
Case 9	76	Male	44	30.4	20. 1/19.6	Productive cough	*Streptococcus pneumoniae*	TBM/COPD/lung adenocarcinoma	Stable
Case 10	88	Male	58	58.7	30.4/28.4	Hemoptysis	*Pseudomonas aeruginosa*	TBM/COPD/bronchiectasis	Stable
Case 11	80	Male	57	31.7	21.0/20.5	Hemoptysis	*Pseudomonas aeruginosa*	TBM/bronchiectasis	Stable

**FIGURE 2:**
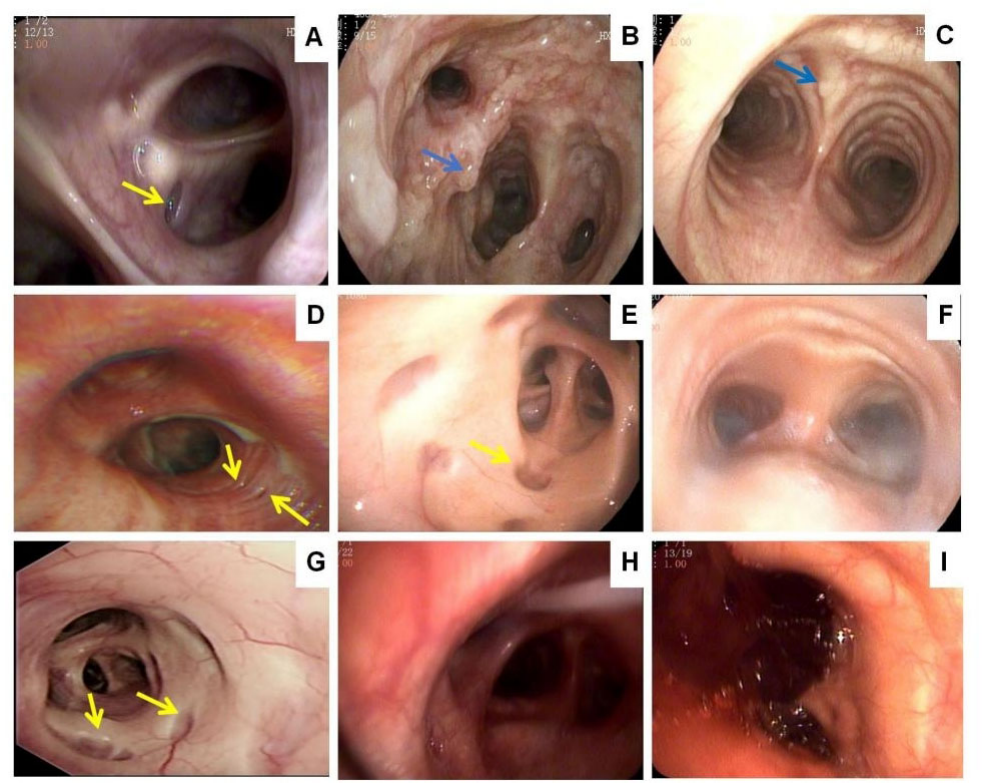
Bronchoscopy: diverticula in the wall of the trachea (**A,D,E,G**, yellow arrow). Bony nodules protruding into the lumen from the submucosa of the tracheobronchial tree (**B,C**, blue arrow). Purulent secretions can be seen in the lumen of the trachea and bronchi **(F,H)**. Formation of blood clots in the trachea **(I)**.

Subsequently, sputum culture and targeted next-generation sequencing were conducted on sputum or BALF samples. *Pseudomonas aeruginosa* (27.3%, *n* = 3) and *Streptococcus pneumoniae* (27.3%, *n* = 3) were the most common pathogens, followed by *Klebsiella pneumoniae* (18.2%, *n* = 2) and *Aspergillus* (18.2%, *n* = 2). Case 6 tested positive for *Mycobacterium tuberculosis*, although the patient showed no symptoms (Table 1). Nine patients underwent bronchoscopy, which confirmed their diagnoses (Figure 2). Diverticula were present in four cases (Figures 2A, D, E, G).

**FIGURE 3:**
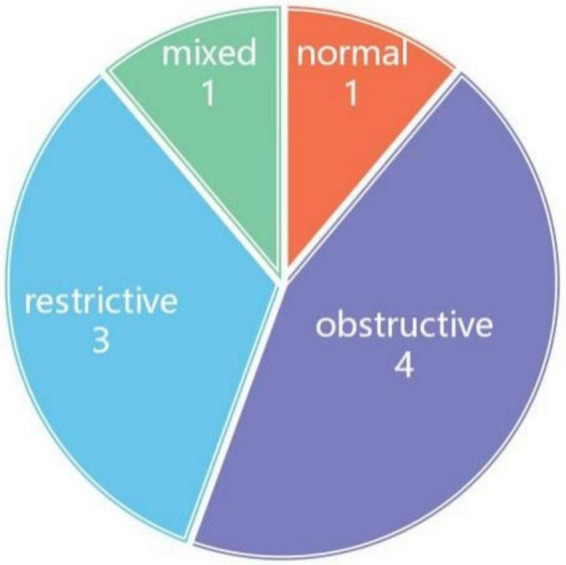
Pulmonary function tests (GOLD Spirometry Guide 2025 was used for classification).

PFTs revealed obstructive patterns in four patients, restrictive patterns in three, and a mixed pattern in one (Figure 3).

Nine patients received antibiotic therapy for recurrent pulmonary infections and/or bronchiectasis exacerbations, 10 patients were treated with mucolytics, and 11 patients received respiratory physiotherapy. Unfortunately, one patient (Case 5) succumbed to respiratory failure and eventually died.

## Discussion

Tracheobronchomegaly (TBM) or Mounier-Kuhn syndrome (MKS), first described in 1932 (2), is a rare disorder characterized by dilation of the trachea and main bronchi due to the pathological arrangement of smooth muscle fibers (5). In general, the terms TBM and MKS have been used interchangeably in the literature, with an estimated 360 published cases until 2013 (4). With improvements in medical technology and an increase in the number of CT scans performed, an additional 109 patients were diagnosed with TBM between 2013 and 2024. To date, fewer than 500 cases of TBM have been reported worldwide (6). Consistent with previous studies, TBM predominantly affects males (7). Wang et al. reported a 5.5:1 male-to-female ratio of (8). The ratio calculated by our team was 4.9:1 (9). Our findings confirmed a striking male predominance, as all the patients were male.

The etiology of this condition remains unclear. Currently, there are two predominant theoretical frameworks: congenital origin via autosomal recessive inheritance (10) and acquired pathogenesis from the loss of elastic fiber-induced airway remodeling (11). An increasing number of people recognize TBM as an acquired condition associated with numerous chronic muscular, respiratory, and autoimmune diseases. These include pulmonary fibrosis, bronchiectasis, emphysema, chronic bronchitis, amyotrophic lateral sclerosis (12), chronic progressive histoplasmosis, giant cell arteritis (13), ankylosing spondylitis, rheumatoid arthritis, primary tracheobronchial amyloidosis (14), Sjogren’s syndrome (15), and homocystinuria (16), which are conditions found in patients with critical illnesses that require prolonged treatment. Hyperinflation of endotracheal or tracheostomy tube cuffs has been observed in a range of patient groups, including those with acute respiratory distress syndrome and respiratory failure (17). In our study, none of the patients showed familial clustering, comorbidities with connective tissue disorders, or tracheal tube insertion, all of which were occasionally associated with TBM.

The clinical presentations of TBM vary widely, ranging from asymptomatic to typical symptoms, including fever, dry cough, sputum production, hemoptysis, chest pain, and dyspnea. Recurrent respiratory infections can result in respiratory failure or death. Compared with other reports of lower hemoptysis rates, our study had a hemoptysis rate of up to 27.3%. Frequent comorbid manifestations included bronchiectasis (45.5%) and recurrent pneumonia (81.8%, *n* = 9). Few patients maintain a lifelong symptom-free status, as in case 4, who was diagnosed during a routine physical examination. Interestingly, the duration of symptoms varied from acute (1 day) to chronic (8 years), reflecting the heterogeneous clinical course of TBM. This variability is likely due to differences in the degree of airway involvement and the presence of comorbidities, such as bronchiectasis, COPD, and ILD. These findings are consistent with those of previous reports showing that TBM often coexists with other pulmonary diseases, complicating clinical management and prognosis. Case 2 presented with a unique combination of TBM and TO, suggesting a potential pathophysiological link that warrants further exploration.

Radiologically, the mean tracheal diameter in our cohort was 32.4 mm, ranging from 30.4 to 62.3 mm, which is consistent with the established diagnostic criteria for TBM. Diverticula were observed in 36.4% of the patients, predominantly in the paratracheal region. This is a common feature of TBM, which results from the weakening of the tracheal wall. Additionally, other radiological findings, such as osteocartilaginous nodules in TO and ILD, were noted in a few cases, indicating a broad spectrum of associated abnormalities in TBM.

PFTs revealed obstructive patterns in 36.4% of the patients, restrictive patterns in 26.3%, and mixed patterns in 9.1%, reflecting the complex pathophysiology of TBM. Impaired mucociliary clearance, airway dilation, and recurrent infections likely contribute to the obstructive features, whereas fibrosis and restrictive changes may result from prolonged disease and inflammation. These findings highlight the importance of regular pulmonary function monitoring in patients with TBM, as the early identification of functional abnormalities can guide management strategies.

The treatment of TBM is primarily supportive, with a focus on managing symptoms and preventing complications. In our cohort, nine patients received antibiotic therapy for recurrent infections, and mucolytics and respiratory physiotherapy were used in 10 and 11 patients, respectively, to improve airway clearance and respiratory function. Notably, one patient showed a reduction in tracheal diameter following treatment, suggesting that appropriate interventions can positively influence the disease course. However, managing hemoptysis may require more invasive approaches, such as vascular interventional therapy or surgical intervention, owing to the risk of recurrent infections and airway bleeding. Timely respiratory support is crucial for patients with respiratory failure.

Despite optimized management, progressive respiratory insufficiency may still occur, necessitating invasive interventions, such as airway stenting or tracheobronchoplasty, to address severe dynamic airway collapse. Although experience with these procedures in TBM cohorts remains limited, reports suggest variable efficacy and a relatively high incidence of complications, including infection and stent migration (18, 19).

Lung transplantation is indicated in patients with end-stage respiratory failure who are unresponsive to conventional therapeutic interventions (20). In addition, efforts should be made to improve the early detection of advanced disease stages. Biomarker discovery and validation could play a crucial role in this regard, as they would allow for more timely identification of patients at a high risk of progressive respiratory insufficiency. This would facilitate earlier intervention and potentially improve patient prognosis. Investigations are needed to elucidate the optimal indications, treatment parameters, and long-term safety profiles of endoscopic laser ablation. Comparative studies with other treatment modalities would also be beneficial in determining its relative efficacy in different patient subsets.

Moreover, given the limited experience with airway stenting and tracheobronchoplasty in TBM cohorts, multicenter collaborative research is essential. This would pool data from multiple institutions and enable a more comprehensive analysis of the efficacy and complication rates of invasive procedures. Such research could also contribute to the establishment of standardized guidelines for their use, minimize variability in treatment approaches, and improve patient outcomes.

The psychological and quality-of-life aspects of patients with TBM should not be overlooked. Supportive care services, including pulmonary rehabilitation, psychological counseling, and patient education, should be integrated into the overall treatment plan. This holistic approach would not only address physical symptoms but also enhance patients’ overall well-being and ability to cope with the disease. As the syndrome remains relatively rare and it is unlikely that any large-scale, systematic study will be conducted in the near future, the authors of this review suggest that future publications should attempt to standardize reported clinical findings. This is with a view to decreasing the wide variability observed in previous case reports.

## Conclusion

This study enhances our understanding of the clinical, radiological, and functional features of TBM or MKS, emphasizing the importance of early diagnosis and a multidisciplinary approach for its management. The high incidence of recurrent respiratory infections and variability in pulmonary function highlight the need for aggressive management to prevent disease progression and improve patient outcomes. Further research is needed to establish standardized treatment protocols and to better define the long-term prognosis of patients with this rare and complex disorder.

## Data Availability

The original contributions presented in this study are included in this article/supplementary material, further inquiries can be directed to the corresponding authors.
